# Does globalization and energy usage influence carbon emissions in South Asia? An empirical revisit of the debate

**DOI:** 10.1007/s11356-022-24457-9

**Published:** 2022-12-22

**Authors:** Bosede Ngozi Adeleye, Darlington Akam, Nasiru Inuwa, Henry Tumba James, Denis Basila

**Affiliations:** 1 Department of Accountancy, Finance and Economics, University of Lincoln, Lincoln, UK; 2 Department of Economics, University of Lagos, Lagos, Nigeria; 3 Department of Economics, Gombe State University, Gombe, Nigeria; 4 Department of Economics, Adamawa State University, Mubi, Nigeria; 5 Department of Accounting, Adamawa State University, Mubi, Nigeria

**Keywords:** Carbon emissions; Energy use, Globalization, Kuznets hypothesis, South Asia

## Abstract

The 2030 United Nations Sustainable Development Goal (SDG) 13 agenda hinges on attaining a sustainable environment with the need to “take urgent action to combat climate change and its impacts”. Hence, this study empirically revisits the debate on the effect of nonrenewable energy and globalization on carbon emissions within the framework of the Kuznets hypothesis using an unbalanced panel data from seven South Asian countries (Bangladesh, Bhutan, India, Maldives, Nepal, Pakistan, and Sri Lanka) covering 1980–2019. The variables of interest are carbon emissions measured in metric tons per capita, energy use measured as kg of oil equivalent per capita, and globalization index. To address five main objectives, we deploy four techniques: panel-corrected standard errors (PCSE), feasible generalized least squares (FGLS), quantile regression (QR), and fully modified ordinary least squares (FMOLS). For the most part, the findings reveal that the (1) inverted U-shaped energy-Kuznets curve holds; (2) U-shaped globalization-Kuznets curve is evident; (3) inverted U-shaped turning points for nonrenewable energy are 496.03 and 640.84, while for globalization are 38.83 and 39.04, respectively; (4) globalization-emission relationship indicates a U-shaped relationship at the median and 75th quantile; and (5) inverted U-shaped energy-Kuznets holds in Pakistan but a U-shaped nexus prevails in Nepal and Sri Lanka; inverted U-shaped globalization-Kuznets holds in Bangladesh and Sri Lanka, but U-shaped nexus is evident in Bhutan, Maldives, and Nepal. Deductively, our results show that South Asia countries (at early stage of development) are faced with the hazardous substance that deteriorates human health. Moreover, the non-linear square term of the nonrenewable energy-emissions relationship is negative, which validates the inverted U-shaped EKC theory. Overall, the effect of energy and globalization on carbon emissions is opposite while the consistency at the 75th quantile result indicates that countries with intense globalization are prone to environmental degradation.

## Introduction

The drive to maintain a sustainable environment necessitated the 2030 United Nations Sustainable Development Goal (SDG) 13 agenda, which is to “take urgent action to combat climate change and its impacts”. Therefore, to address climate change, it becomes imperative to understand its contributing factors: one of which is carbon dioxide (CO_2_) emissions. The sources of carbon emissions are mainly from the burning of fossil fuel in a productive system with active power generation, transport, residential, and industrial sectors (IPCC 2018﻿). These type of carbon emissions are known as “greenhouse” gases which should have been absorbed by space but are trapped by absorbing solar energy thereby heating the earth to cause global warming (National Geographic 2019). This trapping of heat is known as the “greenhouse effect” which exemplifies environmental degradation.

Two key facts emanate from the illustrated scenario. First, the burning of fossil fuel evidences the usage of nonrenewable energy which is divided into four components: coal, natural gas, oil, and nuclear energy. This combination not only alters the earth’s atmosphere but also emit varieties of pollutants that negatively affect human health (Atasoy 2017; IPCC 2018; Barua et al. 2022). Secondly, the burning of fossil fuel typifies an active, vibrant, and growing economy typified by extensive openness with the rest of the world. In other words, nonrenewable energy, economic growth, and globalization are the identified drivers of carbon emissions (Dogan and Aslan 2017; Shahbaz and Sinha 2018; Murshed and Dao 2020; Parker and Bhatti 2020; Adeleye et al. 2021b; Eregha et al. 2021; Shakib et al. 2022; Awan and Azam 2022). Hence, with the danger posed by increasing emissions, most developing economies are primarily saddled with the seemingly impossible task of curbing emissions amidst achieving steady economic growth and unhindered energy supply.

On the link between emissions and globalization, climate change is precipitated by the enormous amount of carbon dioxide produced by the use of renewable and nonrenewable energy sources in a bid to produce goods and services for man’s use (Akadiri et al. 2020﻿; Liu et al. 2020; Rahman 2020; Aluko et al. 2021; Mughal et al. 2021﻿). Climate change is a global phenomenon with its negative consequences affecting directly or indirectly every nation, depending on the level of development of a given nation. It appears nature is lending support to advocates of globalization, though in the negative. Globalization, in the positive sense, promises less restriction in the movement of people, capital, goods and services, technology and culture etc., from one country to another enhancing harmony among people globally (Dong et al. 2018; Sasana et al. 2018﻿; Bataka 2021). Though, Rahman (2020) and Farooq et al. (2022) report that globalization affects the quality of the environment in various ways. Such as increase in trade and production increases carbon dioxide emission, it also allows for transfer of technology, which does reduce carbon dioxide emission in the long run, and further, it supports national economic transformation from agrarian to the desired services economy, the eco-friendly system.

To situate the discourse, this study also contributes to the actualization of the Paris Agreement, sometimes referred to as the Paris Accords or the Paris Climate Accords. The Agreements was adopted by 196 parties in 2015 and borders on climate change mitigation, adaptation, and finance. Empirical support for climate change mitigation and environmental sustainability are diverse. However, several studies suggest that environmental sustainability can be achieved with the reduction in the level of carbon emissions via energy efficiency (Murshed et al. 2022a; Khan et al. 2022), renewable energy use (Murshed et al. 2022b; Hamid et al 2022a), nuclear energy adoption (Nathaniel et al. 2021a, b), globalization (Jahanger et al. 2022; Rehman et al. 2022a), and aggregate domestic consumption (Chishti et al. 2022).

This study positions on South Asia based on four reasons: (1) pollution, (2) economic growth, (3) energy demand, and (4) members of an economic cooperation that focuses on trade. Firstly, according to IQAir (2019), South Asia is the most polluted region, with 27 of the 30 most polluted cities located therein. India inhabits 21 of those cities. For PM_2.5_^1^ using a weighted population average, Bangladesh emerges as the most polluted country followed by Pakistan, Mongolia, Afghanistan, and India with deviations of less than 10% from one another. Among others, the surge in air pollution has adversely affected human health and tourist inflows with negative revenue and socio-economic shocks and spillovers (TERI 2019). Second, the World Bank (2019a, 2019b) positioned the region as the fastest-growing region in the world though growth moderated from 7.2% in 2017 to 6.9% in 2018. Also, the countries have divergent economic outlooks which make comparativeness intrinsic. From United Nations (2019), in contrast to Pakistan, the economic conditions in Bangladesh, Bhutan, and India are mostly positive with positive GDP growth projections. Lastly, energy demand is higher in Asia and projected to double between 2018 and 2050, making it both the largest and the fastest-growing region in the world for energy consumption (EIA 2019). Besides, India is one of the world’s fastest-growing economies during much of the past decade, and they remain primary contributors to future growth in world energy demand (IEA 2019b, 2019a).

The purpose of this study is to contribute to the empirical debate on whether nonrenewable energy use and globalization contribute to carbon dioxide emissions in South Asia and which has the largest significant impact. To achieve this, an unbalanced panel data comprising carbon dioxide emissions per capita, nonrenewable energy per capita, globalization index, and a set of control variables from 1980 to 2019 is used. Study objectives are fivefold: (1) investigate whether the energy-Kuznets curve is evident; (2) test if the globalization-Kuznets hypothesis holds; (3) determine the turning points; (4) examine if there are significant changes across the non-normal distribution of carbon emissions; and (5) evaluate if the energy-Kuznets and globalization-Kuznets curves significantly differ across the countries. To achieve these objectives, we deploy a blend of four estimation techniques which serve as robustness checks: the panel-corrected standard errors (PCSE), feasible generalized least squares (FGLS), quantile regression (QR), and fully modified ordinary least squares (FMOLS) techniques. To the best of knowledge, this is the first study to adopt this approach. For the most part, our results are consistent. We find that energy use exerts an inverted U-shaped relation to carbon emissions while that of globalization indicates a U-shaped relation with emissions. There are also significant differences across countries. The rest of the study is structured as follows: the “Review of extant literature” section reviews the literature; the “Data and empirical strategy” section outlines the data and model; the “Results and discussions” section interprets and discusses the results, while the “Conclusion and policy recommendations” section concludes with policy recommendations.

## Review of extant literature

Carbon emission is the proxy for environmental degradation, and the quest for a sustainable environment led to investigations on the drivers of carbon emissions. Several studies which will be highlighted in this section have explored different factors that aggravate environmental degradation with diverse outcomes partly due to the scope under coverage, the analytical technique, and the choice of control variables. Therefore, without claim to being exhaustive, the carbon emissions literature is reviewed along with time series and panel data frameworks.

### Time series outcomes

Covering the period 2005 to 2016, Ma et al. (2019) use a non-parametric procedure on the relation between carbon emissions and energy consumption to conclude that economic growth is the primary predictor of carbon emissions in China. This outcome is similar to Lin and Raza (2019) who find that energy intensity reduces carbon emissions in Pakistan for the period 1978 to 2017. Likewise, Shaheen et al. (2020) use the ARDL technique to show that in the long run, energy consumption and gross domestic product (GDP) intensify carbon emissions in Pakistan from 1972 to 2014. Salahuddin et al. (2019) use Zivot-Andrews’ breakpoint analysis to conclude that urbanization and globalization are the drivers of emissions in South Africa from 1980 to 2017. Similarly, Wang et al. (2019a, b) on China from 1995 to 2014 use the ridge analysis to reveal the population, urbanization, and industrialization fuel carbon emissions. From 1970 to 2017, Sarkodie and Strezov (2018) deploy the dynamic autoregressive distributed lag (ARDL) model to reveal that energy consumption is a positive predictor of carbon emissions in Australia. Using the ARDL approach on Nigeria from 1981 to 2017, Okoye et al. (2021) provide evidence on energy-led growth.

On Iran from 2002 to 2013 and using the dynamic ordinary least squares (DOLS) and vector error correction model (VECM) procedures, Shabani and Shahnazi (2019) conclude that growth, energy use, and information technology are the drivers of carbon emissions. Also, Li et al. (2019) use the fixed and random-effects models to conclude that urbanization, agro-tech, and information technology are the major drivers of carbon emissions. On the study of Saudi Arabia from 1990 to 2015, Omri et al. (2019) use the fully modified ordinary least squares (FMOLS) and DOLS to conclude that trade openness, financial development, and foreign direct investment (FDI) are the principal contributors of carbon emissions. In the same vein, Yang et al. (2018) use the ARDL technique to find that trade openness and urbanization drive emissions in China from 1995 to 2014. Sarkodie and Strezov (2018) conclude using FMOLS and DOLS that renewable energy stalls emissions in Australia from 1974 to 2013. Shahbaz et al. (2018) also use the ARDL approach to find that globalization, energy use, and economic growth exacerbate emissions in Japan from 1970 to 2014.

On Kuwait, Salahuddin et al. (2018) deploy the ARDL, VECM, and DOLS techniques to find that the principal determinants of carbon emissions from 1980 to 2013 are FDI, energy use, and economic growth. Zhou et al. (2018) find that FDI induces more carbon emissions in China from 2003 to 2015 using the ARDL technique. Roy et al. (2017) deploy the ridge technique on India from 1990 to 2016 and conclude that energy use reduces carbon emissions. Mirza and Kanwal (2017) from the VECM and ARDL procedures find a feedback relation between emissions/growth and emissions/energy use in Pakistan from 1979 to 2009. However, Bukhari and Waseem (2017) find a one-way causal impact from energy use to emission in Pakistan from 1972 to 2013 using the ARDL approach. Also, using the bounds and Bayer and Hanck (2013) cointegration techniques, Okoro et al. (2021) show that economic growth induces gas flaring activities in Nigeria. Likewise, Khan et al. (2018) deploy the FMOLS technique to find that financial development reduces emissions in India, Bangladesh, and Pakistan from 1980 to 2014.

From 1980 to 2020, Alam (2022) finds that trade and economic growth in Bahrain have negative relationship with environmental deterioration. Murshed et al. (2022c) reveal that improving the use of renewable electricity curbs carbon emissions in Argentina from 1971 to 2014. On the study of Oman, Alam et al. (2022) show that energy consumption and financial development are positive determinants of emissions in Oman from 1972 to 2019, while Hamid et al. (2022b) reveal that foreign direct investment inflows, economic growth, and capital investments boost carbon dioxide emissions from 1980 to 2019. Similarly, other time series studies found significant socio-economic indicators exacerbating environmental degradation. Amongst such are Pakistan (Rehman et al., 2022b), Saudi Arabia (Ozturk et al. 2022a), Turkey (Ozturk et al. 2022b), and China (Zhou et al 2022) to mention a few.

### Panel data outcomes

Eurostat (2020) from the study of 27 member countries finds that carbon emission is a major contributor to global warming and account for some 80% of all human-made European Union (EU) greenhouse gas emissions. Shahbaz et al. (2019a, b) deployed cross-correlation techniques to find that globalization reduces carbon emissions from the study of 87 countries from 1970 to 2012. The study validates the existence of the pollution haven hypothesis (PHH), for the FDI/emissions and growth/emissions relations. Adeleye et al. (2021a) use a sample of seven South Asian countries from 1990 to 2019 and three empirical techniques to provide evidence that economic growth and nonrenewable energy exacerbate carbon emissions while renewable energy exert emissions-reducing properties. Neagu and Teodoru (2019) use the DOLS and FMOLS procedures to show that a long-term equilibrium relationship exists among growth, energy use, and greenhouse gas (GHG) emissions in 25 EU countries. Churchill et al. (2019) on the study of G7 countries from 1870 to 2014 use a non-parametric approach to show that emissions and research and development exhibit time-varying features. Nguyen and Kakinaka (2019) deployed panel cointegration techniques in 107 countries from 1990 to 2013 to show that renewable energy stalls carbon emissions in high-income countries. Correspondingly, Shahbaz et al. (2019a, b) use the generalized method of moments (GMM) technique to examine the association between FDI and carbon emissions for the Middle East and North African (MENA) region in 1990–2015.

From the analysis of high, middle, and lower-income countries, Azizalrahman and Hasyimi (2019) use the ARDL procedure to find that urbanization and energy consumption are the drivers of carbon emissions in high-income countries from 1973 to 2013. Using the quantile-on-quantile technique, Mallick et al. (2019) find that the poor do not contribute to carbon emissions in the case of BRICS member-states from 1980 to 2014. Alike, Inglesi-Lotz (2018) uses a non-parametric procedure on the study of BRICS member-states from 1990 to 2014 and finds that carbon emission is reduced with changes in energy and carbon intensity. This outcome is similar to Mahalik et al. (2018) who find that, except for Brazil, coal consumption drives carbon emission in BRICS member-states from 1980 to 2013. Equally, using a non-parametric approach, Chang et al. (2019) on analysing 121 countries show that population surge, energy consumption, economic growth, and carbon intensity are the principal drivers of emissions from 2000 to 2014. Acheampong (2018) deploys the GMM and panel vector autoregressive (PVAR) techniques on a study of 116 countries from 1990 to 2014 to show that feedback causal relation exists between emissions/growth and emissions/energy use. Also, on a study of 17 countries from 1971 to 2013, Sarkodie (2018) deployed the fixed and random-effects techniques to show both globalization and energy usage Granger-cause carbon emissions. On the study of 13 Asian countries from 1980 to 2010, Salim et al. (2017) deploy the augmented mean group (AMG) approach to show that trade liberalization, urbanization, and renewable energy consumption reduce carbon emissions.

### Carbon emissions and globalization (mixed studies)

Furthermore, empirical analyses on globalization and carbon dioxide emissions have revealed inconsistent outcomes which are attributable to differences in empirical techniques, variables selection/combination, geographical scope, and timeframe, among others. For instance, He et al. (2021) find that globalization decreases carbon dioxide emissions in the top 10 energy transition economies. Likewise, Rahman (2020) finds inverse relationship between carbon dioxide emissions and globalization among top 10 electricity consuming countries. Similarly, Liu et al. (2020﻿) report inverted U-shaped relationship between carbon emissions and globalization for G7 countries. U-shape relationship confirms the outcome of positive environmental quality in the long run. Afridi et al. (2019) report trade openness; a proxy of globalization has a negative impact on carbon emissions in among South Asian Association for Regional Cooperation (SAARC) economies. Equally, Asongu et al. (2020) report modulating effect of globalization (trade openness) on carbon dioxide emissions in 44 Sub-Saharan African (SSA) countries. Acheampong et al. (2019) find foreign direct investment mitigating carbon dioxide emissions in SSA. To the contrary, Jun et al. (2021) find a positive association between globalization and carbon dioxide emissions in selected South Asian economies. Also, Rafindadi and Usman (2019) find globalization increasing carbon dioxide emissions in the short run in South Africa, while it reduces it in the long run. Haseeb et al (2018) find negative and insignificant relationship between globalization and carbon dioxide emissions in Brazil, Russian Federation, India, China, and South Africa (BRICS), but Saud et al. (2018) find negative and significant relationship between globalization and energy demand, which is the main driver of carbon dioxide emissions; again the result shows unidirectional causality between globalization and energy consumption in China. On the other hand, Yuping et al. (2021) report evidence of globalization reducing carbon emissions, and both globalization and renewable energy consumption jointly reduce emissions in Argentina. Findings from Cao et al. (2021﻿) reveal that globalization reduces carbon dioxide emissions in OECD countries. Wang et al. (2019a, b) also report unidirectional causality from globalization to carbon dioxide emissions in OECD countries. Khan et al. (2021a, b) examine the position of the USA to find that globalization reduces environmental quality by enhancing carbon dioxide emissions. Lastly, Islam et al. (2021) report globalization having negative effect on carbon dioxide emission in Bangladesh. These empirical findings further reveal inadequate geographical spread of study focus covering South Asian region (see Jun et al. 2021), notwithstanding, the significance of the areas in relation to the effect of globalization and environmental degradation, with a population of over a billion people.

## Data and empirical strategy

### Data

This study employs an unbalanced annual panel data of selected seven South Asian^2^ countries from 1980 to 2019. The dependent variable is carbon emissions *(CO*_*2*_*)* measured in metric tons per capita. The main independent variables are energy use *(ENU)* measured as kg of oil equivalent per capita and globalization index (*GLB*). Since the conjecture is that energy use and globalization contribute to rising carbon emissions (Acheampong et al. 2019; Afridi et al. 2019; Asongu et al. 2020; Adeleye et al. 2021a, 2021b; Ansari et al. 2021), we expect positive coefficients. There are four control variables with direct relations to carbon emissions: gross domestic product per capita *(PC)* at constant 2010 US$ (Chontanawat 2020; Murshed et al. 2020; Adedoyin et al. 2021; Nathaniel et al. 2021a; Akam et al. 2021; Shaari et al. 2021﻿), population growth (*PGR*) (Bhat 2018﻿; Işık et al. 2019; Azizalrahman and Hasyimi 2019; Salahuddin et al. 2019; Adedoyin and Bekun 2020; Yasin et al. 2020), renewable energy (*REN*) (Feng and Chen 2018﻿; Balsalobre-Lorente et al. 2018; Sharif et al. 2019; Khan et al. 2020; Rahman and Velayutham 2020), and regulatory quality (*RQ*) (Biswas et al. 2012; Chen et al. 2018; Zakaria and Bibi 2019﻿; Dada et al. 2021; Psychoyios et al. 2021). The variable names, description, sources, and expected signs, are presented in Table 1.

**Table 1:** Variables, description, and signs

Variable	Description	Signs	Sources
CO2	CO2 emissions (metric tons per capita)	N/A	World Bank (2020﻿)
PC	GDP per capita (constant 2010 US$)	+	World Bank (2020)
PGR	Population growth (annual %)	+	World Bank (2020)
REN	Renewable energy consumption (% of total final energy consumption)	−	World Bank (2020)
RQ	Regulatory quality: estimate (from − 2.5 to 2.5)	−	World Bank (2020)
ENU	Energy use (kg of oil equivalent per capita)	+	World Bank (2020)
GLB	KOF globalization index (from 0 to 100)	+	Gygli et al. (2019); Dreher (2006); Potrafke (2015)

Source: Authors’ compilations

## Summary statistics and correlation analysis

Table 2 presents the descriptive statistics (using untransformed values) and pairwise correlation (using the natural logarithms) among the variables. From the result, Maldives has the highest CO_2_ emissions (1.453) with a maximum and minimum value of 3.038 and 0.278, respectively. The high CO_2_ emissions of Maldives comparatively can be traceable to the burning of fossil fuels for energy and cement production (Shukla et al. 2016; Afridi et al. 2019; Ritchie and Roser 2020). This implies that the Maldives is involved more in production activities than any other South Asian country. India and Pakistan rank second and third on average CO_2_ emissions given their industrialization drives. The CO_2_ emissions of Bangladesh and Nepal are the lowest with an average of 0.242 and 0.114, respectively. This could be traceable to the slow rate of industrialization in both countries. This supposition aligns with Afridi et al. (2019). The performance of South Asian countries in terms of nonrenewable energy (ENU) showed that Maldives and Pakistan are the highest energy users, while Bangladesh and Bhutan are the lowest. This can be explained by rising demand for energy orchestrated by industrialization in the Maldives and Pakistan. Lastly, Sri Lanka, India, and Pakistan have the highest globalization index (GLB) of 49.4%, 45.6%, and 45.1%, respectively, which implies that globalization is driving economic growth in these countries but very weak in Bangladesh, Bhutan, Maldives, and Nepal.

**Table 2:** Summary statistics and pairwise correlation analysis

Variable		CO2	PC	PGR	REN	RQ	ENU	GLB
Full sample	Observation	259	264	280	182	168	185	266
	Mean	0.65	1581.07	1.945	57.245	− 0.439	349.425	39.902
	Stand. dev	0.604	1819.268	0.925	28.709	0.43	138.157	11.227
	Minimum	0.028	280.899	− 0.36	0.903	− 1.169	104.862	17.642
	Maximum	3.038	8209.465	4.567	95.92	1.027	892.087	62.254
Bangladesh	Observation	37	40	40	26	24	35	38
	Mean	0.242	615.392	1.858	53.971	− 0.905	147.917	37.689
	Minimum	0.096	359.456	1.042	34.747	− 1.127	104.862	23.563
	Maximum	0.533	1287.821	2.686	73.16	− 0.797	229.246	52.235
Bhutan	Observation	37	39	40	26	24	5	38
	Mean	0.566	1378.941	1.645	91.92	− 0.613	277.925	28.602
	Minimum	0.054	390.065	− 0.36	86.905	− 1.169	105.453	22.793
	Maximum	1.712	3128.001	3.211	95.92	0.033	366.986	42.657
India	Observation	37	40	40	26	24	35	38
	Mean	0.977	975.897	1.734	48.435	− 0.35	413.112	45.575
	Minimum	0.449	422.904	1.015	36.021	− 0.553	286.164	30.888
	Maximum	1.818	2169.14	2.329	58.653	− 0.156	636.57	62.254
Maldives	Observation	37	25	40	26	24	5	38
	Mean	1.453	6604.668	3.106	2.197	0.109	701.35	38.925
	Minimum	0.278	4599.638	1.733	0.903	− 0.48	228.076	31.287
	Maximum	3.038	8209.465	4.567	4.459	1.027	892.087	50.448
Nepal	Observation	37	40	40	26	24	35	38
	Mean	0.114	480.81	1.669	89.766	− 0.61	333.093	34.005
	Minimum	0.028	280.899	− 0.267	84.375	− 0.849	301.627	17.642
	Maximum	0.334	859.024	2.729	95.12	− 0.353	434.449	48.98
Pakistan	Observation	37	40	40	26	24	35	38
	Mean	0.724	854.993	2.632	49.981	− 0.639	422.591	45.121
	Minimum	0.411	552.616	2.029	44.276	− 0.905	317.211	33.666
	Maximum	0.988	1197.913	3.364	58.091	− 0.482	500.432	55.346
Sri Lanka	Observation	37	40	40	26	24	35	38
	Mean	0.477	2035.588	0.97	64.442	− 0.063	390.353	49.394
	Std. dev	0.236	990.439	0.355	6.259	0.207	76.163	9.143
	Minimum	0.204	909.325	0.129	52.876	− 0.422	301.579	37.387
	Maximum	1.102	4011.682	1.656	78.087	0.276	551.021	60.9
Pairwise correlation	lnCO2	1.000						
	lnPC	0.783***	1.000					
	PGR	0.023	− 0.034	1.000				
	REN	− 0.685***	− 0.637***	− 0.491***	1.000			
	RQ	0.352***	0.547***	0.083	− 0.449***	1.000		
	ENU	0.650***	0.711***	− 0.152**	− 0.448***	0.635***	1.000	
	GLB	0.593***	0.505***	− 0.351***	− 0.364***	0.006	0.590***	1.000

***p < 0.01, **p < 0.05; *ln* natural logarithm, *CO2* carbon emissions, *PC* GDP per capita, *PGR* population growth, *REN* renewable energy, *RQ* regulatory quality, *ENU* nonrenewable energy, *GLB* globalization index

Source: Authors’ computations

From the pairwise correlation reported on the lowest panel of Table 2, all the variables with the exception of renewable energy indicate significant positive association with carbon missions. Among the regressors, there is no evidence of perfect linear representation given all the correlation statistics are below 0.80 from multicollinearity becomes a concern.

### Empirical strategy

Following our study objectives, we start with specifying a baseline model with carbon emissions expressed as a linear function of control variables which are per capita GDP, population growth, renewable energy, and regulatory quality:

1 $$\mathrm{ln}{{CO}_{2}}_{it}={\beta }_{0}+{\beta }_{1}{\mathrm{ln}PC}_{it}+{\beta }_{2}{PGR}_{it}+{\beta }_{3}{REN}_{it}+{\beta }_{4}{RQ}_{it}+{\varepsilon }_{it}$$

To achieve the first objective of investigating whether the energy-Kuznets curve is present, we include both the level and square of nonrenewable energy into Eq. (1):

2 $$\mathrm{ln}{{CO}_{2}}_{it}={\beta }_{0}+{\beta }_{1}{\mathrm{ln}PC}_{it}+{\beta }_{2}{PGR}_{it}+{\beta }_{3}{REN}_{it}+{\beta }_{4}{RQ}_{it}+{\gamma }_{1}{ENU}_{it}+{\gamma }_{2}{ENUSQ}_{it}+{v}_{it}$$

To realize the second objective on the evidence of globalization-Kuznets curve in South Asia, both the level and squared terms of the globalization index are incorporated into Eq. (1):

3 $$\mathrm{ln}{{CO}_{2}}_{it}={\beta }_{0}+{\beta }_{1}{\mathrm{ln}PC}_{it}+{\beta }_{2}{PGR}_{it}+{\beta }_{3}{REN}_{it}+{\beta }_{4}{RQ}_{it}+{\varphi }_{1}{GLB}_{it}+{\varphi }_{2}{GLBSQ}_{it}+{\tau }_{it}$$

To ascertain if the energy-Kuznets and globalization-Kuznets hypotheses hold despite the inclusion of all the variables under evaluation, Eqs. (2) and (3) are infused into Eq. (1) to become

4 $$\mathrm{ln}{{CO}_{2}}_{it}={\beta }_{0}+{\beta }_{1}{\mathrm{ln}PC}_{it}+{\beta }_{2}{PGR}_{it}+{\beta }_{3}{REN}_{it}+{\beta }_{4}{RQ}_{it}+{\gamma }_{1}{ENU}_{it}+{\gamma }_{2}{ENUSQ}_{it}+{\varphi }_{1}{GLB}_{it}+{\varphi }_{2}{GLBSQ}_{it}+{\eta }_{it}$$

where ${CO}_{2}$ is the per capita CO2 emissions. $ENU$ is the nonrenewable energy per capita. $ENUSQ$ is the square of nonrenewable energy per capita. $GLB$ is the globalization index. $GLBSQ$ is the square of globalization index. The control variables are as follows: $PC$ is the real per capita GDP. $PGR$ is the population growth. $REN$ is the renewable energy. $RQ$ is the regulatory quality. ${\beta }_{i}, {\gamma }_{i}, {\varphi }_{i}$ are the parameters to be estimated; ${\varepsilon }_{it}, {v}_{it}, {\tau }_{it, } {\eta }_{it}$ are the error terms. To evaluate if the energy-Kuznets and globalization-Kuznets curve significantly differ across the countries, Eqs. (2) and (3) are modified to allow for country-level analysis where the subscripts denote the country *i* and the time period *t* (1980–2019).

To address the core objectives of the study, Eqs. (2) and (3) assume similarity for the parameters ${\gamma }_{1}$, ${\gamma }_{2}$, ${\varphi }_{1}$, and ${\varphi }_{2}$ which depend neither on a specific country nor on the time period. It is assumed that all countries take on the same shape of the functional relation of the emissions-energy and emissions-globalization paradox. More importantly, Eqs. (2) and (3) permit the evaluation of different forms of emissions-energy and emissions-globalization relationships. For instance, with respect to the energy-Kuznets hypothesis, (i) ${\gamma }_{1}<0$, ${\gamma }_{2}>0$ reveals a U-shaped relationship; (ii) ${\gamma }_{1}>0$, ${\gamma }_{2}<0$ reveals an inverse U-shaped relationship, representing the EKC. The energy turning point of this curve is computed by $\widehat{\tau }=\left(0.5{}^{{\widehat{\gamma }}_{1}}\!\left/ {}_{{\widehat{\gamma }}_{2}}\right.\right)$; (iii) ${\gamma }_{1}>0$, ${\gamma }_{2}>0$ reveals a monotonically increasing linear relationship; (vi) ${\gamma }_{1}<0$, ${\gamma }_{2}<0$ reveals a monotonically decreasing linear relationship; and (vii) ${\gamma }_{1}=0$, ${\gamma }_{2}=0$ reveals a level relationship. In general, the turning point is when the first derivative of Eq. (2) with respect to energy use is equated to zero. Analogous depiction for Equation [3] for the globalisation-Kuznets hypothesis. For the expected a priori, ${\beta }_{1},{\beta }_{2}>0$ and ${\beta }_{3},{\beta }_{4}<0$. Also, to ensure that this turning point is within the minimum and maximum values of energy use and globalization index, that is, within the observed range of the data, both variables are estimated in their *level* forms, while other variables are transformed into their natural logarithms with the exception of the population growth rate and regulatory quality.

### Estimation approach

The empirical analysis starts with the application of the cross-sectional dependence test among the countries to determine the suitable methods to apply. The risk of cross-sectionally dependent panels is very high due to the close proximities of the units and given the possibility of sharing common features. In the event of cross-sectional dependence (CSD) in the data, biased estimates and inferences will occur (Pesaran 2004). To forestall such, the study engages the Pesaran (2004, 2007)^3^ test for cross-sectional dependency (CD) which can be applied to small and large panels. The null hypothesis of no CSD which can be rejected at the 1%, 5%, and 10% significance levels is expressed as

5 $$CD=\sqrt{2T/N\left(N-N\right)}\left({\textstyle\sum_{i=1}^{N-1}}{\textstyle\sum_{k=i+1}^N}{\widehat\rho}_{i,k}\right)$$

In the event of cross-sectional dependence, the data is subjected to second-generation unit root tests to avoid spurious results. The cross-sectional augmented Im, Pesaran, and Shin (CIPS) developed by Pesaran (2007) is engaged. The CIPS test, which is the augmented variant of Im et al. (2003) unit root test, is expressed as

6 $$CIPS\left(N,T\right)= \overline{T }={N}^{-1}\sum\nolimits_{i=1}^{N}{t}_{i}\left(N,T\right)$$

where $N$ and $T$ are the numbers of cross-sections and years, respectively. The left-hand side of Eq. (6) is the unit root test for heterogeneous panels, while on the right-hand side, the term ${t}_{i}$ is the ordinary least squares (OLS) *t*-ratios employed in cross-sectional averaged augmented Dickey-Fuller (ADF) regression. As a preliminary check, we also used the Maddala and Wu (1999) first-generation unit root test which assumes cross-sectional independence. Thereafter we assess whether a long-run relationship exists among the variables using the second-generation panel cointegration tests proposed by Westerlund (2007). This technique is suitable in the presence of CSD in the data, and the null hypothesis of no cointegration can be rejected at the 1%, 5%, and 10% significance levels. Finally, given the presence of cross-sectional dependence in the data and cointegration among the variables, the Prais-Winsten regression model with panel-corrected standard errors (PCSE) which also controls for heteroscedasticity and serial correlation is used to estimate all the models. For robustness checks and to observe the consistency of the results, we deploy the feasible generalized least squares (FGLS) techniques. Given that both PCSE and FGLS techniques pertain to only the conditional mean of the dependent variable, we re-estimate Eqs. (2), (3), and (4) using the bootstrap simultaneous quantile regression (QR). Quantile analysis provides intrinsic information across the distribution of the dependent variable (Koenker and Bassett 1978; Koenker and Hallock 2001). The method fits a regression line through the conditional quantiles of a distribution and allows the investigation of the relationship between regressors across different parts of the distribution of the dependent variable.

## Results and discussions

### Pre-estimations: CSD, PURT, and cointegration

The results from pre-estimations are presented in Table 3. The results of the Pesaran (2004) CD test reject the null hypothesis of no cross-sectional dependence at the 1% significance level suggesting that any shock in one country may be transmitted to other South Asian countries. To examine the stationarity of the variables, the Maddala and Wu (1999) first-generation unit root test which assumes “cross-sectional independence” and Pesaran (2007) second-generation unit root test that presumes “cross-sectional dependency” are deployed. The outcomes from both tests indicate that all the variables with the exception of PGR are stationary after the first difference. The Westerlund (2007) cointegration results indicate a long-run association among the variables exist.

**Table 3:** CSD, panel unit root, and cointegration tests

Variables	Pesaran (2004) CD	Maddala and Wu (1999)		Pesaran (2007) CIPS	
		Level	1st Diff	Level	1st Diff
lnCO2	26.088***	4.624	200.17***	0.941	− 7.70***
lnPC	26.316***	2.084	75.21***	− 0.773	− 4.14***
PGR	12.676***	11.37	47.27***	− 5.003***	N/A
REN	20.218***	6.167	164.65***	1.085	− 5.91***
RQ	4.841***	15.294	158.19***	− 0.454	− 3.63***
ENU	16.677***				
GLB	26.18***	16.855	57.29***	1.816	− 3.32***
Westerlund (2007) cointegration test					
Variance ratio = − 1.869**					

***p < 0.01, **p < 0.05; *ln* natural logarithm, *N/A* not applicable, *CO2* carbon emissions, *PC* real GDP per capita, *PGR* population growth, *REN* renewable energy, *RQ* regulatory quality, *GLB* globalization index; energy use excluded during panel unit root testing due to 87.5% loss of observations from Bhutan and Nepal. Both countries have 5 data points each (see Table 2 on Summary Statistics); one-period lag is used for panel unit root test of PGR for Pesaran (2007) CIPS; panel unit root test performed using the multipurt routine in Stata16. Source: Authors’ computations

### Estimations: full sample (PCSE, FGLS, and QR)

With each as robustness checks, Table 4 shows the result from estimating Eqs. (1)–(2) using the PCSE (columns [1] to [4]) and FGLS (columns [5] to [8]) techniques. We restrict interpretations to the variables that address the core of the study—nonrenewable energy and globalization index.

**Table 4:** PCSE and FGLS results, full sample (Dep Var: lnCO2)

Variables	PCSE, Main Analysis				FGLS, Robustness			
	[1]	[2]	[3]	[4]	[5]	[6]	[7]	[8]
lnPC	0.703*** (21.35)	0.570*** (4.286)	0.657*** (30.88)	0.465*** (4.958)	0.626*** (9.641)	0.248*** (2.857)	0.691*** (11.28)	0.695*** (7.326)
PGR	0.105*** (3.715)	0.124** (2.203)	0.218*** (4.465)	− 0.0606 (− 0.859)	0.137*** (3.016)	0.0818** (2.150)	0.138*** (2.619)	0.111 (1.417)
REN	− 0.00849*** (− 7.854)	− 0.0256*** (− 9.256)	− 0.00681*** (− 4.920)	− 0.0377*** (− 6.075)	− 0.00658*** (− 2.902)	− 0.0255*** (− 13.10)	− 0.00542** (− 2.244)	− 0.0189*** (− 4.804)
RQ	− 0.314*** (− 5.593)	− 0.401*** (− 2.755)	− 0.288*** (− 4.335)	− 0.460*** (− 3.133)	− 0.107 (− 1.518)	− 0.0756 (− 1.052)	− 0.195** (− 2.329)	− 0.609*** (− 4.976)
ENU		0.00837*** (11.57)		0.0115*** (5.175)		0.00596*** (9.883)		0.00763*** (6.284)
ENUSQ		− 8.44e − 06*** (− 7.597)		− 1.17e − 05*** (− 4.286)		− 4.65e − 06*** (− 5.344)		− 7.45e − 06*** (− 6.051)
GLB			− 0.116*** (− 3.543)	− 0.158** (− 2.418)			− 0.100*** (− 3.125)	− 0.127*** (− 2.935)
GLBSQ			0.00150*** (3.885)	0.00146** (2.368)			0.00128*** (3.651)	0.00128*** (2.889)
Turning point		496.03	38.83			640.84	39.04	
Constant	− 5.374*** (− 18.82)	− 5.346*** (− 4.660)	− 3.270*** (− 5.141)	− 0.224 (− 0.123)	− 4.928*** (− 8.578)	− 2.644*** (− 3.921)	− 3.675*** (− 3.668)	− 3.509** (− 2.106)
Observations	140	103	140	103	140	103	140	103
R-squared	0.710	0.883	0.774	0.899				
Countries	7	7	7	7	7	7	7	7
Wald statistic	1463	3176	1891	9333	234.1	1355	401.4	644.0

***p < 0.01, **p < 0.05, *p < 0.1; *z*-statistics in (); *ln* natural logarithm; − 8.44e − 06 = 8,440,000.00; *PC* GDP per capita, *PGR* population growth, *REN* renewable energy, *RQ* regulatory quality, *ENU* nonrenewable energy, *GLB* globalization index

Source: Authors’ computations

**Fig. 1:**
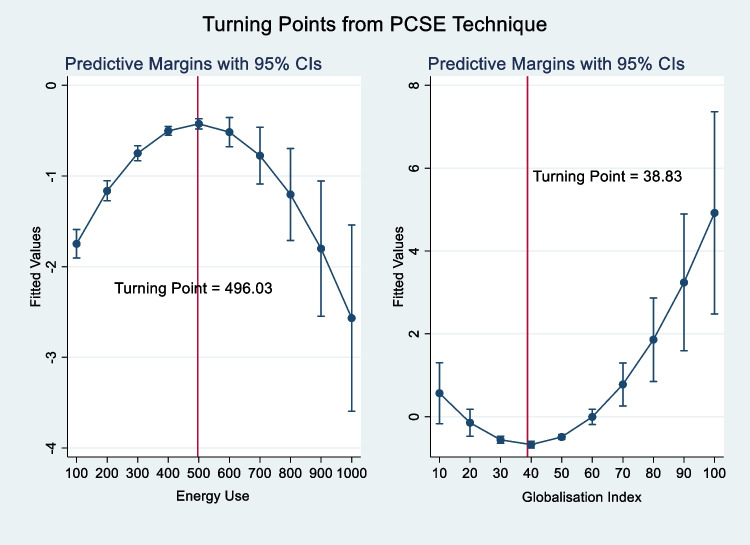
Turning points of nonrenewable energy and globalization index from PSCE technique. Source: Authors’ computations

**Fig. 2:**
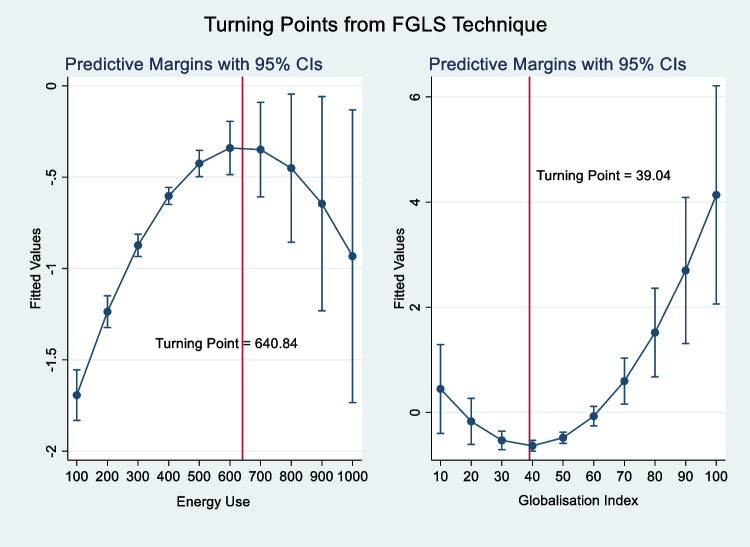
Turning points of nonrenewable energy and globalization index from FGLS technique. Source: Authors’ computations

As expected, nonrenewable energy upsurges environmental pressure in South Asia with a statistically significant relationship ranging from 1 to 5%, on average, in the long run. This is an indication that a unit increase in nonrenewable energy use contributes positively to carbon emissions by 0.6 to 1.2% (see columns [2], [4], [6], and [8]). The result is similar to Nasreen et al. (2017﻿), Hunjra et al. (2020﻿), and Adeleye et al. (2021a) who found that being reliant on nonrenewable energy sources for meeting energy demand in South Asia induces poor environmental quality (Murshed 2021; Murshed et al. 2022d). Thus, South Asian countries (at their early stage of development) are faced with the hazardous substance that deteriorates human health. Moreover, the non-linear square term of the nonrenewable energy-emissions relationship is negative, which validates the inverted U-shaped EKC theory as shown in Figs. 1 and 2. This is an implication that South Asian countries attain a threshold point at which the deteriorating impact of dirty energy began to diminish. Besides, the turning point occurs between 496.03 and 640.84 kg of oil equivalent per capita. This can be attributed to the population’s awareness of cleaner energy and its importance in improving quality of life (Sarkodie 2018). This finding is in contrast to Chunyu et al. (2021﻿) who found a positive monotonic energy-emissions relationship.

On the contribution of GLB, a unit increase in globalization will decrease carbon emissions between 10 and 15.8% (see columns [3], [4], [7], and [8]). The result in this sense surmises that opening markets through trade, finance, and foreign direct investment reduced the adverse effect of carbon emissions in South Asia. Thus, the transfer of clean energy and better regulatory strategies from trade liberalization can improve a quality environment (Acheampong et al. 2019; Alvarado et al. 2022; Deng et al. 2022; Azam and Raza 2022; Azam et al. 2022). In line with previous studies (Chang et al. 2019; Rafindadi and Usman 2019; Zaidi et al. 2019﻿), the result indicates that globalization helps mitigate environmental harm. However, the square term of the globalization-emissions relationship is positive, which invalidates the EKC theory. As indicated in Figs. 1 and 2, the threshold point occurs between 38.8 and 39 KOF globalization index, suggesting that at 38.8% and 39% turnaround point, carbon emission begins to rise. The implication of this is that at the early stage of development, South Asia enjoys environmental sustainability through globalization but declines after the threshold point is attained. Thus, at the early stage, the result supports the *halo effect hypothesis*; thereafter, *the pollution haven hypothesis* sets in. Moreover, the positive increasing effect may be due to the relocation of polluting firms from high-income countries with quality regulation to low-income countries with less stringent environmental regulation (Doytch and Uctum 2016﻿; Chang et al. 2019). The environmental pressure of globalization is unsurprising because the South Asian countries in this study are non-high-income countries with increased pollution. Contrarily, Acheampong et al. (2019) found a negative linear and non-linear globalization-emissions relationship. The discrepancy in the result could be attributed to the choice of globalization proxy.

Next, Table 5 presents the estimates using simultaneous quantile regression (QR)^4^ across three quantiles (*Q* = 0.25, *Q* = 0.50, *Q* = 0.75); the estimated results are shown for energy use, globalization, and energy use + globalization. First, with respect to energy use in models [2 and 4], the results reveal an inverted U-shaped EKC relationship. This implies that energy use from dirty technology significantly increases carbon emissions, thus, deteriorating South Asia’s environment. The result aligns across the three quantiles but with a larger impact at 75th quartile suggesting that carbon emission increase for countries with more energy use. Moreover, the squared term shows that at the turnaround point, the deteriorating impact begins to fizzle out**.** However, the globalization-emission relationship indicates a U-shaped relationship (for *Q* = 50 and *Q* = 75 in the model [3] and *Q* = 75 in the model [4]), implying that at the early stage of development, globalization mitigates environmental quality; thereafter, deterioration sets after a threshold point. The consistency at the 75th quantile result indicates that countries with high trade liberalization and foreign direct investment are prone to environmental degradation at development. Also, consistent with the PCSE and FGLS results is a significant contribution to the literature as it indicates that the use of nonrenewable energy and globalization without strong environmental regulation will infer poor environmental quality in South Asian countries.

**Table 5:** Simultaneous quantile results, full sample (Dep Var: lnCO2)

Variables	Energy use			Globalization			Energy use + globalization		
	*Q* = 0.25	*Q* = 0.50	*Q* = 0.75	*Q* = 0.25	*Q* = 0.50	*Q* = 0.75	*Q* = 0.25	*Q* = 0.50	*Q* = 0.75
lnPC	0.219 (1.192)	0.110 (0.544)	0.137 (0.335)	0.666*** (3.943)	0.768*** (4.213)	0.701*** (3.823)	0.157 (0.915)	0.267 (1.408)	0.308** (1.994)
PGR	− 0.0237 (− 0.202)	− 0.0598 (− 0.707)	0.157 (0.757)	0.0521 (0.546)	0.376*** (2.830)	0.366*** (3.273)	− 0.00598 (− 0.0357)	− 0.112 (− 1.016)	− 0.216*** (− 3.045)
REN	− 0.0351*** (− 8.536)	− 0.0366*** (− 6.228)	− 0.0295*** (− 2.902)	− 0.0132*** (− 3.968)	8.19e-05 (0.0199)	0.00254 (0.353)	− 0.0364*** (− 3.290)	− 0.0415*** (− 5.186)	− 0.0488*** (− 10.09)
RQ	− 0.144 (− 0.977)	− 0.0349 (− 0.238)	− 0.174 (− 0.430)	− 0.312 (− 1.460)	− 0.134 (− 0.899)	− 0.0323 (− 0.147)	− 0.0382 (− 0.210)	− 0.305 (− 1.246)	− 0.164 (− 1.026)
ENU	0.00907*** (4.388)	0.00834*** (7.805)	0.00966*** (5.022)				0.00865** (2.195)	0.0113*** (3.330)	0.0140*** (6.742)
ENUSQ	− 8.36e − 06*** (− 3.564)	− 7.74e − 06*** (− 5.559)	− 8.71e − 06*** (− 3.573)				− 8.05e − 06* (− 1.821)	− 1.09e − 05*** (− 2.729)	− 1.40e − 05*** (− 5.293)
GLB				− 0.101 (− 0.818)	− 0.135** (− 2.114)	− 0.0811** (− 1.983)	− 0.0322 (− 0.346)	− 0.173 (− 1.636)	− 0.291*** (− 4.416)
GLBSQ				0.00122 (0.922)	0.00186*** (2.630)	0.00128*** (3.442)	0.000379 (0.459)	0.00156* (1.704)	0.00255*** (4.129)
Turning Point	542.46	538.76	554.54		36.29	31.68			
Constant	− 2.440* (− 1.787)	− 1.215 (− 0.706)	− 2.369 (− 0.659)	− 3.188 (− 1.080)	− 4.486* (− 1.926)	− 5.113* (− 1.910)	− 1.163 (− 0.409)	1.965 (0.888)	5.265*** (3.255)
Observations	103	103	103	140	140	140	103	103	103

***p < 0.01, **p < 0.05, *p < 0.1; *t*-statistics in (); *ln* natural logarithm; − 8.36e − 06 = 8,360,000.00; *PC* GDP per capita, *PGR* population growth, *REN* renewable energy, *RQ* regulatory quality, *ENU* nonrenewable energy, *GLB* globalization index

Source: Authors’ computations

### Estimations: country-level (FMOLS)

Since panel analysis beclouds individual cross-sectional dynamics, Table 6 displays the country-level results from the FMOLS technique.^5^ Country-level results are mixed. Restricting discussions to nonrenewable energy and globalization, the effect of nonrenewable energy consumption is positive and significant in Pakistan suggesting that an increase induces a rise in emissions by 0.00748% (Zafar et al. 2019; Pham et al. 2020; Khan et al. 2021a, b). However, the coefficient of the squared term is negative and significant signifying an inverted U-shaped relationship. Thus, the EKC holds in Pakistan with the turning point at 442.60 kg of oil equivalent per capita, suggesting that when the nonrenewable energy consumption reaches 442.60 kg of oil equivalent per capita, CO2 emissions decline. The opposite holds for Nepal and Sri Lanka. At the initial level, the effect of nonrenewable energy consumption is negative and significant which indicates that the consumption indices less carbon emissions. This might be attributable to the use of energy efficient technologies coupled with strengthening their environmental policies at the earlier stages of development. But further use of nonrenewable energy sources (with turning point for Nepal at 385.42 and Sri Lanka at 306.39) causes emissions to rise. Hence, a U-shaped nexus prevails in both countries supporting the findings of Ben Jebli and Ben Youssef (2015﻿﻿).

**Table 6:** Country-level results from FMOLS technique (Dep Var: lnCO2)

Variables	Bangladesh		Bhutan	India		Maldives	Nepal		Pakistan		Sri Lanka	
	[1]	[2]	[3]	[4]	[5]	[6]	[7]	[8]	[9]	[10]	[11]	[12]
lnPC	0.0304 (0.16)	0.440*** (9.62)	0.0996 (1.64)	0 (0.00)	0.317*** (5.11)	0.0791** (2.66)	1.728*** (7.20)	− 0.954*** (− 5.13)	0 (0.00)	0.419*** (6.75)	0.0164 (0.46)	0.443*** (17.31)
PGR	− 0.00820 (− 0.14)	0.237*** (8.19)	− 0.0306 (− 1.52)	− 0.0257 (− 0.28)	− 0.113 (− 1.61)	− 0.0386*** (− 8.57)	0.194*** (5.64)	− 0.0473 (− 1.88)	− 0.208*** (− 20.36)	0.00417 (0.11)	0.399*** (9.12)	− 0.0415 (− 1.68)
REN	− 0.0317*** (− 5.39)	− 0.0254*** (− 16.25)	− 0.149*** (− 40.09)	− 0.0146* (− 2.48)	− 0.0265*** (− 9.57)	− 0.450*** (− 66.34)	− 0.0931*** (− 24.98)	− 0.0899*** (− 15.06)	− 0.0255*** (− 18.05)	− 0.0140*** (− 9.34)	0.000474 (0.11)	− 0.0249*** (− 10.54)
RQ	− 0.0991* (− 2.51)	0.00990 (0.42)	− 0.0916*** (− 6.48)	0.0299 (1.26)	0.0318 (1.46)	− 0.0107 (− 1.40)	− 0.370** (− 3.23)	− 0.479*** (− 3.76)	0.156*** (15.30)	− 0.0124 (− 0.64)	− 0.0518* (− 2.30)	0.0715* (2.42)
ENU	− 0.000138 (− 0.02)			0.00206 (1.04)			− 0.0259*** (− 6.84)		0.00748** (2.81)		− 0.00815*** (− 3.71)	
ENUSQ	0.00000356 (0.27)			− 0.000000503 (− 0.35)			0.0000336*** (6.93)		− 0.00000845** (− 2.99)		0.0000133*** (5.29)	
GLB		0.108*** (9.14)	− 0.100*** (− 6.01)		− 0.0109 (− 0.84)	− 0.0683*** (− 5.38)		− 0.310*** (− 9.02)		− 0.0319 (− 1.36)		0.232*** (8.18)
GLBSQ		− 0.00106*** (− 8.86)	0.00145*** (6.62)		0.0000367 (0.28)	0.000892*** (5.98)		0.00430*** (9.72)		0.000402 (1.80)		− 0.00204*** (− 7.50)
Turning Point		50.94	34.48			38.28	385.42	35.67	442.60		306.39	56.86
Constant	0 (0.00)	− 5.785*** (− 10.79)	14.20*** (27.57)	0 (0.00)	− 0.164 (− 0.28)	2.151*** (5.53)	0 (0.00)	17.16*** (11.26)	0 (0.00)	− 1.798 (− 1.64)	0 (0.00)	− 8.908*** (− 11.71)
Observations	18	19	19	18	19	19	18	19	18	19	18	19

***p < 0.01, **p < 0.05, *p < 0.1; *t*-statistics in (); *ln* natural logarithm; − 8.36e − 06 = 8,360,000.00; *PC* GDP per capita, *PGR* population growth, *REN* renewable energy, *RQ* regulatory quality, *ENU* nonrenewable energy, *GLB* globalization index; country-level analyses performed using the fmols cointreg routine in Stata16

Source: Authors’ computations

On globalization, at the initial, a percentage increase in globalization triggers emissions by 0.108% and 0.232% in Bangladesh and Sri Lanka, respectively (Shahbaz et al. 2016; Kassouri and Altintas 2020; Wang et al. 2020; Rahman 2020; Yurtkuran 2021). But upon reaching a threshold of 50.94 and 56.86, respectively, the effect causes emissions to decline. The inverted U-shaped nexus supports the globalization-EKC hypothesis which is consistent with He et al. (2021), and Xiaoman et al. (2021). The plausible explanation is that globalization allows for green technology transfer across nations, thereby reducing the use of pollution-provoking resources which in turn improve environmental quality. To the contrary, globalization induces a negative effect on emissions in Bhutan, Maldives, and Nepal at the initial stage implying that globalization indices environmental quality. This outcome supports the earlier findings of Shahbaz et al. (2016), Rahman (2020), and Rafindadi and Usman (2019) who found that globalization causes less pollution of the environment. The plausible explanation for this result is not farfetched. As an economy converges through globalization, they have more access to adopt advanced technologies and technical knowledge which allows the utilization of energy efficiently. But, upon attaining a threshold of 34.48, 38.28, and 35.67, respectively, carbon emissions start to rise causing a degradation of the economy. These results corroborate the finding of Wang et al. (2020) for G7 countries that globalization stimulate environmental degradation.

## Conclusion and policy recommendations

The relationship among carbon dioxide emissions, nonrenewable energy, and globalization has fuelled recent debates. As such, this study contributes to the discourse by engaging an unbalanced panel data of seven South Asian countries (Bangladesh, Bhutan, India, Maldives, Nepal, Pakistan, and Sri Lanka) covering 1980–2019. Using a blend of robust econometric techniques from panel-corrected standard errors (PCSE), feasible generalized least squares (FGLS), quantile regression (QR), and fully modified ordinary least squares (FMOLS) techniques, our results provide sufficient evidence to address the study objectives. We find that (1) the inverted U-shaped energy-Kuznets curve is evident in the full sample; (2) a U-shaped globalization-Kuznets curve is prevalent from the full sample; (3) the inverted U-shaped turning points are 496.03 and 640.84 for nonrenewable energy, while for globalization are 38.83 and 39.04, respectively; (4) we find significant changes across the non-normal distribution of carbon emissions; and (5) inverted U-shaped energy-Kuznets holds in Pakistan, but a U-shaped nexus prevails in Nepal and Sri Lanka; inverted U-shaped globalization-Kuznets holds in Bangladesh and Sri Lanka, but U-shaped nexus is evident in Bhutan, Maldives, and Nepal.

### Scientific contributions

Relative to existing studies, this study contributes significantly to the literature in five strategic areas. First, it shows that the energy-EKC holds given the presence of increased globalization. Second, it confirms that a U-shaped globalization-emission nexus holds given increased usage of nonrenewable energy. Third, it provides sufficient evidence that intense globalization contributes to environmental degradation. Fourth, it reveals the distinct heterogeneities across the South Asian economies such that some countries respond favourably to energy usage compared to intense globalization, and lastly, it deploys four robust analyses (PCSE, FGLS, FMOLS, and QR) to substantiate findings. To the best of our knowledge, this is the first study to deploy such battery techniques especially the quantile regression (QR) to model energy-globalization-emissions nexus within the EKC framework.

### Policy recommendations

The above findings provide suggestions for policy directives for South Asian government. The detrimental effect of globalization can be mitigated through the promotion of clean and environmentally friendly technologies in the production of goods and services. This is because better and efficient regulatory strategies may warrant manufacturers to adopt green technology causing the abatement of carbon emissions. More so, given that the bulk of carbon emissions are from the transportation sector, it becomes expedient for the government and private sector to prioritize investment and re-engineer the transport system so people can carpool rather than use their individual cars. Since the environment is a public good, promoting green growth through clean innovation and quality environmental regulatory can help save resources and reduce environmental pollution. Furthermore, individual South Asian government may develop its environmental easing policies. Also, the stakeholders should collectively put in place effective energy management in charge of reducing the negative effect of energy-consuming industries and energy-consuming technologies to ensure pollution easing in these countries. Also, having shown the harmful effect of globalization, this study suggests that South Asian countries should increase trading among themselves since they share a common agenda of reducing carbon emissions and enhancing a clean environment. Promoting regional trade through South Asian Association for Regional Cooperation (SAARC) will ensure that green technologies are adopted by manufacturers during the production of goods and services. Lastly, tackling climate change and ensuring a sustainable environment (SDG13) requires that de-carbonization measures be pursued to enable a healthy environment that will reduce health impacts due to energy-related air pollution (SDG3) by 2030. However, there exists a dilemma for developing economies like those of South Asia who may require a trade-off. This is because the drive to pursue economic growth agendas will elicit more carbon emissions which will further degrade the environment. We leave this open for more constructive policy discourse on the quagmire of globalization-led degradation.

## Data Availability

The datasets used during the current study are available from the corresponding author on reasonable request.

## Footnotes

1 Fine particulate matter.

2 Afghanistan is dropped for substantial loss of data points on the three variables of interest.

3 Pesaran (2015) extends the analysis of the Pesaran (2004) CSD test and shows that the implicit null of the test is weak cross-sectional dependence. Interested reader is referred to Sect. 29.7 “Testing for error cross-sectional dependence” in Pesaran (2015﻿) *Time Series and Panel Data Econometrics*, 1Ed., Oxford Press.

4 Due to space, estimation is restricted to Eqs. (2)–(3).

5 Analysis restricted to Eqs. (2) and (3). Energy use models are excluded for Bhutan and Maldives due to 87.5% loss of observations. Both countries have 5 data points each (See Table 2 of Summary Statistics).
